# Risk factors associated with left atrial appendage thrombosis in patients with non-valvular atrial fibrillation by transesophageal echocardiography

**DOI:** 10.1007/s10554-023-02841-x

**Published:** 2023-05-07

**Authors:** Xingpeng Wang, Xiang Xu, Wenting Wang, Haiyun Huang, Feng Liu, Chen Wan, Qing Yao, Huakang Li, Zhihui Zhang, Zhiyuan Song

**Affiliations:** 1 Department of Cardiovascular Medicine, Center for Circadian Metabolism and Cardiovascular Disease, Southwest Hospital, Army Medical University, Chongqing, China; 2 Department of Medical Ultrasonics, Southwest Hospital, Army Medical University, Chongqing, China

**Keywords:** Non-valvular atrial fibrillation, Thrombosis, Left atrial appendage, Transesophageal echocardiography, Predictive model

## Abstract

Purpose: This study investigated possible mechanism of left atrial appendage (LAA) thrombosis and constructed a model to evaluate the future risk of LAA thrombosis and spontaneous echo contrast (SEC) in non-valvular atrial fibrillation (NVAF) patients. Methods: This retrospective study included 2591 patients diagnosed with NVAF. Patients were divided based on the presence of transesophageal echocardiography (TEE) into a thrombus group, SEC group, and control group. General, biochemical, and echocardiography data of the three groups were analyzed. The variables independently associated with LAA thrombosis and SEC were determined by the logistic regression analysis. A nomogram was constituted based on the regression analysis and the discriminatory ability was analyzed by receiver operating characteristic (ROC) curve. Results: LAA thrombosis and SEC were present in 110 (4.2%) patients and 103 (3.9%) patients, respectively. AF type (OR = 1.857), previous stroke (OR = 1.924), fibrinogen (OR = 1.636), diameters of the left atria (OR = 1.094), left ventricular ejection fraction (OR = 0.938), and LAA maximum caliber (OR = 1.238) resulted as independent risk factors for LAA thrombosis and SEC. The area under curve of the nomogram established by multivariate logistic regression was 0.824. Conclusions; Through the study, 6 independent risk factors related to the LAA thrombosis and SEC were found, and an effective nomogram was constructed to predict the LAA thrombosis and SEC in NVAF patients.

## Introduction

Atrial fibrillation (AF) is one of the most common clinical sustained tachyarrhythmias [1]. A recent analysis of the Framingham cohort estimated a prevalence of AF in the general population to range from 0.4 to 1%, which increases with age [2–4], while in the older population, it can reach 7.2% (over 65 years old), and 10.3% (over 75 years and older) [1]. Non-valvular atrial fibrillation (NVAF) is the most common type of AF. Chest discomfort, lightheadedness or feeling faint, a fluttering in the chest, heart palpitations, and shortness of breath are some of the most common symptoms of NVAF.

Previous studies have suggested that the risk of ischemic stroke in NVAF patients is 5 times higher than that in patients with sinus rhythm [5], which is an important cause of stroke. In addition, a series of studies have shown that more than 90% of embolus of thromboembolism complications in NVAF patients come from left atrial appendage (LAA) [6, 7]. Thus, it is believed that ischemic stroke in NVAF patients is closely related to LAA thrombosis. In addition to the loss of atrial systolic function and stasis of blood flow during atrial fibrillation, ischemic stroke in NVAF patients may also be associated with endocardial injury or dysfunction, as well as abnormal coagulation and fibrinolytic function [8]. However, the concrete-related factors and mechanism of LAA thrombosis are still not fully understood.

Transesophageal echocardiography (TEE) is the main way to detect LAA thrombosis and spontaneous echo contrast (SEC) in patients with NVAF, with high specificity and sensitivity. Some previous studies focused on individual factors of the LAA thrombus formation and stroke in patients with NVAF. The present study aimed to investigate factors related to LAA thrombosis and LAA SEC, and integrate various factors to construct a relatively reliable estimation model to predict future risk of LAA thrombosis and SEC in NVAF patients. These data provide basic evidence for clinical prevention and treatment of NVAF patients with LAA thrombosis or thromboembolism complications.

## Methods

### Study design

This is a single-center retrospective study.

### Study subjects

Patients diagnosed with AF who were hospitalized in the cardiovascular department of First Affiliated Hospital of Army Medical University and examined by TEE from August 1, 2014 to May 31, 2021 were included in the study. The inclusion criteria were: (1) patients diagnosed with NVAF by using 2019 AHA/ACC/HRS criteria,[9] which generally refers to AF without moderate-to-severe mitral stenosis (potentially requiring surgical intervention) or in the absence of an artificial (mechanical) heart valve and not imply the absence of valvular heart disease; (2) AF confirmed by electrocardiogram (ECG) or dynamic electrocardiogram (DCG); (3) patients who completed at least once TEE examination as a routine examination on admission;(4) Patients who have not received regular oral-anticoagulation. Patients with uncomplete data were excluded. This study was approved by the Ethical Committee of the First Affiliated Hospital of Army Medical University ((B) KY2021050), June 21, 2021. Informed consent was waived for this was a retrospective study.

### Transesophageal echocardiography (TEE)

**Fig. 1:**
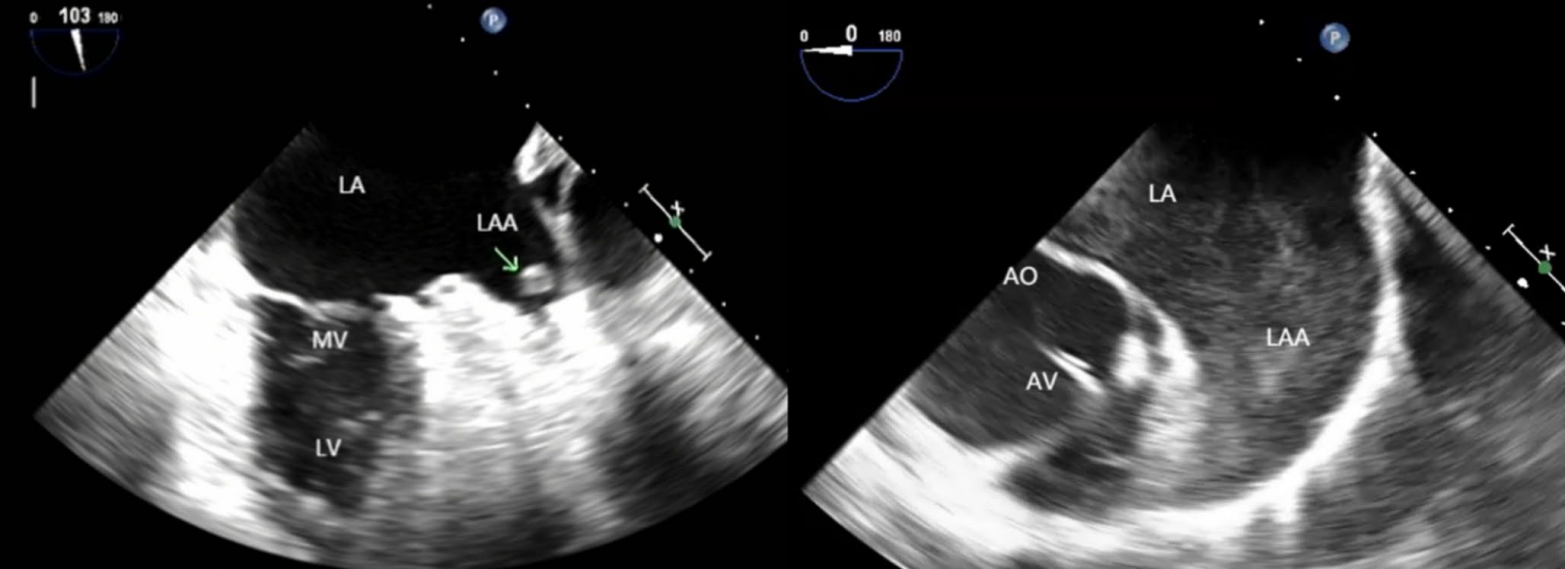
TEE imaging of LAA. Left: arrow showed thrombus. Right: severe SEC could be seen in the LAA and LA. LA: Left atria; LAA: Left auricular appendage; LV: Left ventricular; MV: Mitral valve; AO: aorta

Philips iE 33 color Doppler ultrasonography and X7-2t transesophageal ultrasound probe (frequency 2 ~ 7 MHz) were used in this study. An experienced ultrasonic diagnostician handled the tools. Patients underwent ECG monitoring and ensured that the heart rate was less than 120 bpm during the examination. For patient in AF rhythm, the median values of the measurements in 3 to -5 consecutive cardiac cycles were calculated and recorded. The caliber and depth of LAA were measured from multiple angles, and thrombosis (solid or hypoechoic regiment shadow, Fig. 1) or severe SEC (smoky, very dense blood flow echo signal that can be observed in a regular or even low enhancement[10]**)** in the left atria (LA) and LAA were carefully observed.

### Grouping

Patients were divided based on the presence of LAA and/or LA thrombosis and severe SEC into 3 groups: thrombus group (thrombosis found by TEE), SEC group (no thrombosis but severe SEC found by TEE), and control group (no thrombosis and severe SEC found by TEE).

### Variables

The following data were collected from all patients who were first admitted and did not undergo cardiac interventional surgery: (1) Basic information: age, sex, height, weight, and body mass index (BMI = weight(kg)/height(m) squared); (2) AF types: paroxysmal atrial fibrillation, persistent atrial fibrillation (the paroxysmal AF and persistent AF was defined as abnormal heart rhythm last up no more than a week and more than a week ); (3) Combined diseases: stroke, hypertension, coronary heart disease (CHD), dilated cardiomyopathy (DCM), diabetes; (4) CHA_2_DS_2_-VASc score: The CHA2DS2-VASc score was the sum of points after addition of one point each for heart failure, hypertension, diabetes, vascular disease, age 65–74 years, and female sex and two points each for previous thromboembolism and age ≥ 75 years; (5) Medication history: history of angiotensin-converting enzyme inhibitor (ACEI) or angiotensin receptor blockers (ARB), β-blocker, amiodarone, statin, aspirin, clopidogrel, loop diuretics, spironolactone, digitalis. (6) TTE: the anterior-posterior diameters of the left ventricle (LV), left atria (LA), and right ventricle (RV) in the left ventricle long-axis view, the transverse diameter of the right atria (RA) in the apical 4 chamber view. left ventricular ejection fraction (LVEF) assessed by M-type ultrasound measurements in the left ventricle long-axis view and Simpson’s method in the apical 4 chamber view; (7) TEE: the caliber and depth of LAA at 0°,45°, 90°, 135°. Maximum caliber and depth, and whether there is thrombus or SEC in LA and/or LAA. (8) Erythrocyte count (RBC), neutrophil count (NEUT), lymphocyte count (LN) and platelet count, fibrinogen (fib), prothrombin time (PT), thrombin time (TT), and activated partial thromboplastin time (APTT). (9) Glycosylated hemoglobin, serum creatinine (Scr), glomerular filtration (GFR), cholesterol, high-density lipoprotein cholesterol (HDL-c), low-density lipoprotein cholesterol (LDL-c), and triglyceride.

### Statistical analysis

SPSS 26.0 was used for statistical analysis. Continuous data were tested with the Kolmogorov-Smirnov test for normal distribution. Normally distributed continuous data were expressed as means ± SD. Categorical data are expressed as n (%). Normally distributed continuous data were tested using the student-t test or ANOVA, while non-normally distributed continuous data were analyzed by the Mann-Whitney U test or Kruskal-Wallis test. Categorical data were analyzed by the chi-square test or Fisher’s exact test. And Bonferroni correction was used to correct the p-value of the pairwise comparison. Binary logistics regression was used to analyze the different variables between the thrombosis group/SEC group and the control group and determine the independent risk factors and its OR. A nomogram was construct on the basis of the results of binary logistics regression and was drawn by using the rms package of R, version 3.0 (http://www.r-project.org/). The nomogram is depending on proportionally converting each regression coefficient (β) in logistic regression to a 0 to 100 point rating scale (the effect of the variable with the highest absolute value of β coefficient is determined as 100 points). The points are added across independent variables to calculate total points, which are converted to predicted probabilities. Receiver operating characteristic (ROC) curves were constructed, and the area under the curve (AUC) of this model was calculated to evaluate the discriminant ability. Sensitivity, specificity, were calculated based on the optimal cut-off value. A *P*-value < 0.05 was considered statistically significant.

## Results

### Detection rate of thrombosis and severe SEC

A total of 2591 NVAF patients were included in this study, including 1418 males and 1173 females, with an average age of 63.92 ± 11.35 years old. A total of 110 patients with thrombosis were found by TEE, and the detection rate of thrombosis was 4.2% (95% CI 3.4%-5.0%). Among them, 110 patients (100%) had LAA thrombus, and 1 patient had LA thrombus at the same time (0.9%). In addition, 103 cases had severe SEC, with a detection rate of 4.0% (95% CI 3.2%-4.7%), all located in LAA, among which 55 cases (53.4%) were combined with LA severe SEC.

### Demographic parameters

We then compared clinical characteristics data among different groups (thrombus group, SEC group, and control group) (Table 1). Compared with the control group, patients in the thrombus group and SEC group were older (*p* < 0.001). Moreover, the proportion of persistent or permanent atrial fibrillation, CHA_2_DS_2_-VASc score ≥ 2 (*p* < 0.001) and the proportion of patients with previous stroke and DCM were also higher in the thrombus group and SEC group vs. control group (*p* < 0.005); while there were no significant differences between thrombus group and SEC group (*p* > 0.05). The proportion of patients with CHD in the SEC group was higher than that in the control group (*p* = 0.002); yet, this was not seen when comparing the thrombus group and the control group (*p* > 0.05).

**Table 1:** Demographic variables among groups

Variable		Thrombus group (N = 110)	SEC group (N = 103)	Control group (N = 2378)	*P* value between groups	Pairwise comparison	*P* value
**Age (years)**		66.6 ± 10.7	68.1 ± 9.4	63.6 ± 11.4	＜0.001	Thrombus vs. Control	0.001
						s vs. Control	< 0.001
						Thrombus vs. SEC	0.561
**Gender**	**Male (n/%)**	63 (57.3%)	60 (58.3%)	1295 (54.5%)	0.646		
	**Female (n/%)**	47 (42.7%)	43 (41.7%)	1083 (45.5%)	0.646		
**AF type**	**Persistent or Permanent AF (n/%)**	75 (68.2)	69 (67%)	941 (39.6%)	＜0.001	Thrombus vs. Control	< 0.001
						s vs. Control	< 0.001
						Thrombus vs. SEC	0.853
	**Paroxysmal AF (n/%)**	35 (31.8%)	34 (33%)	1437 (60.4%)	＜0.001	Thrombus vs. Control	< 0.001
						s vs. Control	< 0.001
						Thrombus vs. SEC	0.853
**BMI (Kg/m²)**		24.1 ± 3.3	23.8 ± 3.4	24.4 ± 3.4	0.109		
**CHA**_**2**_ **DS**_**2**_ **-VASc score ≥ 2 (n/%)**		85 (77.3%)	85 (82.5%)	1508 (63.4%)	＜0.001	Thrombus vs. Control	< 0.001
						s vs. Control	< 0.001
						Thrombus vs. SEC	0.34
**Combined decease**	**Stroke (n/%)**	19 (17.3%)	18 (17.5%)	191 (8%)	＜0.001	Thrombus vs. Control	< 0.001
						s vs. Control	< 0.001
						Thrombus vs. SEC	0.969
	**Hypertension (n/%)**	59 (53.6%)	60 (58.3%)	1190 (50%)	0.211		
	**Diabetes (n/%)**	26 (23.6%)	18 (17.5%)	379 (15.9%)	0.097		
	**CHD (n/%)**	74 (67.3%)	80 (77.7%)	1487 (62.5%)	0.005	Thrombus vs. Control	0.315
						s vs. Control	0.002
						Thrombus vs. SEC	0.09
	**DCM (n/%)**	14 (12.7%)	9 (8.7%)	71 (3%)	＜0.001	Thrombus vs. Control	< 0.001
						s vs. Control	0.001
						Thrombus vs. SEC	0.348
**Medication**	**ACEI/ARB**	28 (25.5%)	31 (30.1%)	622 (26.2%)	0.660		
	**β blocker**	31 (28.2%)	39 (37.9%)	755 (31.7%)	0.300		
	**Amiodarone**	9 (8.2%)	7 (6.8%)	179 (7.5%)	0.929		
	**Statin**	32 (29.1%)	32 (31.1%)	605 (25.4%)	0.321		
	**Aspirin**	17 (15.5%)	20 (19.4%)	353 (14.8%)	0.443		
	**Clopidogrel**	14 (12.7%)	6 (5.8%)	244 (10.3%)	0.231		
	**Loop diuretics**	14 (12.7%)	8 (7.8%)	222 (9.3%)	0.415		
	**Spironolactone**	13 (11.8%)	12 (11.7%)	203 (8.5%)	0.287		
	**Digitalis**	8 (7.3%)	4 (3.9%)	123 (5.2%)	0.516		

s: Spontaneous echo contrast; BMI: Body Mass Index; CHD: Coronary atherosclerotic heart disease; DCM: Dilated cardiomyopathy; ACEI: Angiotensin-converting enzyme inhibitor; ARB: Angiotensin receptor blockers

### Complete blood cell (CBC), coagulation function, renal function, and lipid profiles

Compared with the control group, Fib, PT, APTT, glycosylated hemoglobin, and SCr were higher, and GFR was lower in the thrombus group and SEC group (*p* < 0.05), while there was no significant difference between the thrombus group and SEC group (*p* > 0.05). Moreover, NEUT in the thrombus group was significantly higher (*p* < 0.001), while LN and HDL-c were significantly lower than in the control group (*p* < 0.05); still, no significant difference in these variables were found in the SEC group compared with the control group or thrombus group. The RBC, PLT, cholesterol, LDL-c, and triglyceride among the three groups differed to some extent, but the observed differences were not statistically significant. Details can be seen in Table 2.

**Table 2:** Laboratory examination among groups

Variables		Thrombus group (N = 110)	SEC group (N = 103)	Control group (N = 2378)	*P* value between groups	Pairwise comparison		*P* value
**Erythrocyte Count (10** ^**12**^ **/L)**		4.56 ± 0.61	4.45 ± 0.61	4.57 ± 0.58	0.096			
**Neutrophil count (10** ^**9**^ **/L)**		4.48 ± 1.97	3.95 ± 1.57	3.70 ± 1.48	＜0.001	Thrombus vs. Control		< 0.001
						s vs. Control		0.206
						Thrombus vs. SEC		0.142
**Lymphocyte count (10** ^**9**^ **/L)**		1.58 ± 0.66	1.57 ± 0.58	1.69 ± 0.59	0.005	Thrombus vs. Control		0.007
						s vs. Control		0.057
						Thrombus vs. SEC		0.603
**Blood platelet count (10** ^**9**^ **/L)**		170.40 ± 52.50	166.10 ± 66.60	176.90 ± 58.80	0.055			
**Fib (g/L)**		2.90 ± 0.92	3.01 ± 1.06	2.59 ± 0.74	＜0.001	Thrombus vs. Control		< 0.001
						s vs. Control		< 0.001
						Thrombus vs. SEC		0.363
**PT (/s)**		13.84 ± 5.47	13.31 ± 4.14	12.50 ± 5.79	＜0.001	Thrombus vs. Control		< 0.001
						s vs. Control		< 0.001
						Thrombus vs. SEC		0.353
**APTT (/s)**		30.55 ± 6.78	30.44 ± 5.52	29.23 ± 6.06	0.004	Thrombus vs. Control		0.036
						s vs. Control		0.008
						Thrombus vs. SEC		0.636
**TT (/s)**		19.87 ± 13.30	21.39 ± 19.20	19.1 ± 10.00	0.146			
**Glycated hemoglobin (%)**		6.40 ± 1.02	6.60 ± 1.86	6.20 ± 1.17	＜0.001	Thrombus vs. Control		0.005
						s vs. Control		0.003
						Thrombus vs. SEC		0.902
**Scr (µmoI/L)**		81.74 ± 19.50	77.88 ± 17.25	73.64 ± 16.18	＜0.001	Thrombus vs. Control		< 0.001
						s vs. Control		0.037
						Thrombus vs. SEC		0.14
**GFR (ml/min)**		76.10 ± 18.90	78.20 ± 17.70	85.00 ± 18.40	＜0.001	Thrombus vs. Control		< 0.001
						s vs. Control		0.001
						Thrombus vs. SEC		0.417
**Cholesterol (mmol/L)**		4.08 ± 1.12	4.06 ± 1.02	4.18 ± 1.03	0.258			
**HDL-c (mmol/L)**		1.06 ± 0.28	1.13 ± 0.28	1.15 ± 0.29	0.008	Thrombus vs. Control		0.002
						s vs. Control		0.65
						Thrombus vs. SEC		0.065
**LDL-c (mmol/L)**		2.56 ± 0.77	2.52 ± 0.82	2.61 ± 0.74	0.455			
**TG (mmol/L)**		1.43 ± 1.38	1.27 ± 0.74	1.43 ± 0.98	0.080			

s: Spontaneous echo contrast; Fib: Fibrinogen; PT: Prothrombin time; APTT: Activated partial thromboplastin time; Scr: Serum creatinine; GFR: Glomerular filtration rate; HDL-c: High density liptein cholesterol; LDL-c: Low density liptein cholesterol; TG: Triglyeride

### Echocardiography

The TTE and TEE analysis showed that compared with the control group, the LA, RV, LV, and RV in the thrombus group and SEC group were significantly increased (*p* < 0.05), same as the caliber and depth of LAA at 0°,45°, 90°, 135°. Maximum LAA caliber and depth in the thrombus group and SEC group were also significantly increased compared with the control group (*p* < 0.05). Also, LVEF was lower in the thrombus group and SEC group compared with the control group (*p* < 0.005). There were no significant differences in these variables between the thrombus group and the SEC group. Details can be seen in Table 3.

**Table 3:** The transthoracic echocardiography and transesophageal echocardiography parameters among groups

Variables		Thrombus group (N = 110)	SEC group (N = 103)	Control group (N = 2378)	*P* value between groups	Pairwise comparison	*P* value
**TTE**	**LV (mm)**	51.50 ± 7.85	51.10 ± 7.98	48.80 ± 5.51	＜0.001	Thrombus vs. Control	0.002
						s vs. Control	0.005
						Thrombus vs. SEC	0.847
	**LA (mm)**	46.70 ± 5.91	46.80 ± 5.90	41.00 ± 6.94	＜0.001	Thrombus vs. Control	< 0.001
						s vs. Control	< 0.001
						Thrombus vs. SEC	0.957
	**RA (mm)**	45.40 ± 7.81	44.60 ± 6.61	39.4 ± 7.24	＜0.001	Thrombus vs. Control	< 0.001
						s vs. Control	0.001
						Thrombus vs. SEC	0.271
	**RV (mm)**	21.70 ± 4.19	20.80 ± 2.68	20.1 ± 2.54	＜0.001	Thrombus vs. Control	< 0.001
						s vs. Control	< 0.001
						Thrombus vs. SEC	0.652
	**LVEF (%)**	50.80 ± 12.50	51.80 ± 12.50	58.70 ± 9.07	＜0.001	Thrombus vs. Control	< 0.001
						s vs. Control	< 0.001
						Thrombus vs. SEC	0.716
**TEE0°**	**LAA caliber (mm)**	19.90 ± 3.14	19.70 ± 2.70	17.70 ± 2.49	＜0.001	Thrombus vs. Control	< 0.001
						s vs. Control	< 0.001
						Thrombus vs. SEC	0.833
	**LAA depth (mm)**	27.90 ± 3.61	28.20 ± 3.80	25.80 ± 3.56	＜0.001	Thrombus vs. Control	< 0.001
						s vs. Control	< 0.001
						Thrombus vs. SEC	0.866
**TEE 45°**	**LAA caliber (mm)**	19.50 ± 3.00	19.20 ± 2.61	17.20 ± 2.40	＜0.001	Thrombus vs. Control	< 0.001
						s vs. Control	< 0.001
						Thrombus vs. SEC	0.868
	**LAA depth (mm)**	27.90 ± 3.61	28.20 ± 3.77	25.50 ± 3.59	＜0.001	Thrombus vs. Control	< 0.001
						s vs. Control	< 0.001
						Thrombus vs. SEC	0.693
**TEE 90°**	**LAA caliber (mm)**	19.70 ± 3.21	19.70 ± 2.65	17.40 ± 2.51	＜0.001	Thrombus vs. Control	< 0.001
						s vs. Control	< 0.001
						Thrombus vs. SEC	0.679
	**LAA depth (mm)**	28.00 ± 3.85	28.30 ± 3.56	25.40 ± 3.61	＜0.001	Thrombus vs. Control	< 0.001
						s vs. Control	< 0.001
						Thrombus vs. SEC	0.598
**TEE 135°**	**LAA caliber (mm)**	20.10 ± 3.23	20.00 ± 3.10	17.70 ± 2.74	＜0.001	Thrombus vs. Control	< 0.001
						s vs. Control	< 0.001
						Thrombus vs. SEC	0.76
	**LAA depth (mm)**	28.00 ± 3.80	27.60 ± 3.43	25.00 ± 3.43	＜0.001	Thrombus vs. Control	< 0.001
						s vs. Control	< 0.001
						Thrombus vs. SEC	0.632
**TEE Max**	**LAA caliber (mm)**	20.72 ± 3.27	20.56 ± 3.06	18.44 ± 2.75	＜0.001	Thrombus vs. Control	< 0.001
						s vs. Control	< 0.001
						Thrombus vs. SEC	0.887
	**LAA depth (mm)**	29.43 ± 3.96	29.42 ± 3.82	26.58 ± 3.65	＜0.001	Thrombus vs. Control	< 0.001
						s vs. Control	< 0.001
						Thrombus vs. SEC	0.983

TTE: Transthoracic echocardiography; TEE: Transesophageal echocardiography; SEC: Spontaneous echo contrast; LV: Left ventricular; LA: Left atria; RA: Right atria; RV: Right ventricular; LVEF: Left ventricular ejection fraction; LAA: Left auricular appendage

### Multiple regression analysis

**Fig. 2:**
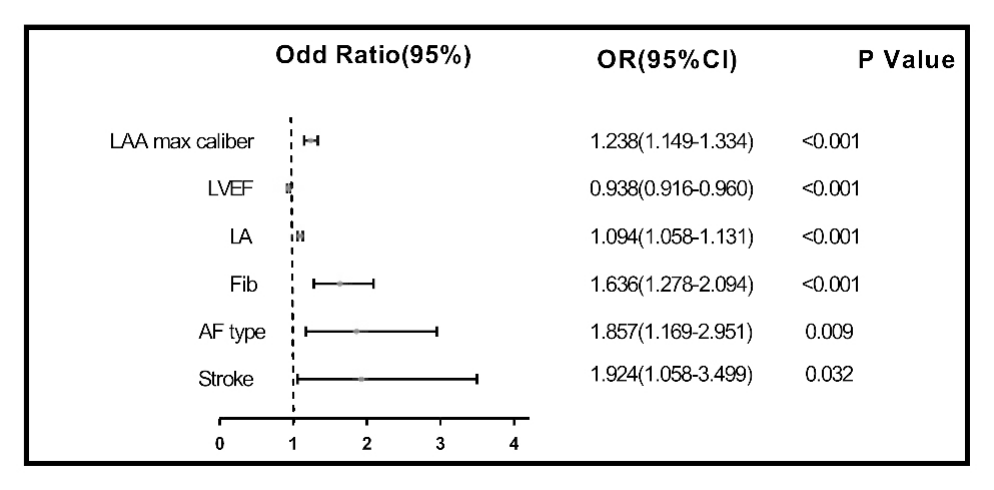
Forest plot of predictors of LAA thrombosis and SEC. LA: Left atria; LAA: Left auricular appendage; LVEF: Left ventricular ejection fraction; AF: Atrial fibrillation; Fib: Fibrinogen

To address the issue of small sample numbers, we combined the thrombus group with the SEC group. Variables found to be remarkably different among the three group in former analyses included age, CHA_2_DS_2_-VASc score, type of atrial fibrillation, previous stroke, diabetes and DCM, Fib, PT, APTT, glycosylated hemoglobin, HDL-c, SCr, GFR, LA, LV, RA, RV, LVEF, LAA max caliber, and depth. All these factors were consequently included in the multivariate model. The results suggested that the AF type (OR = 1.857, 95%CI1.169-2.951, *p* = 0.009), previous stroke (OR = 1.924, 95%CI1.058-3.499, *p* = 0.032), Fib (OR = 1.636, 95%CI1.278-2.094, *p* < 0.001), LA (OR = 1.094, 95%CI1.058-1.131, *p* < 0.001), LVEF (OR = 0.938, 95%CI0.916-0.960, *p* < 0.001), and maximum caliber of LAA (OR = 1.238, 95%CI1.149-1.334, *p* < 0.001) were independent risk factors of LAA thrombosis and SEC in NVAF patients (*p* < 0.05, Fig. 2).

### The nomogram and its estimation ability

**Fig. 3:**
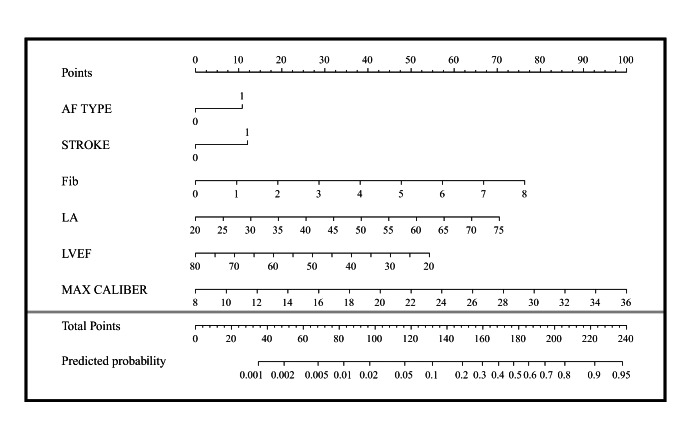
The nomogram to estimate the risk of LAA thrombosis and SEC in NVAF patients. AF: Atrial fibrillation; Fib: Fibrinogen; LAA: Left auricular appendage; LVEF: Left ventricular ejection fraction

**Fig. 4:**
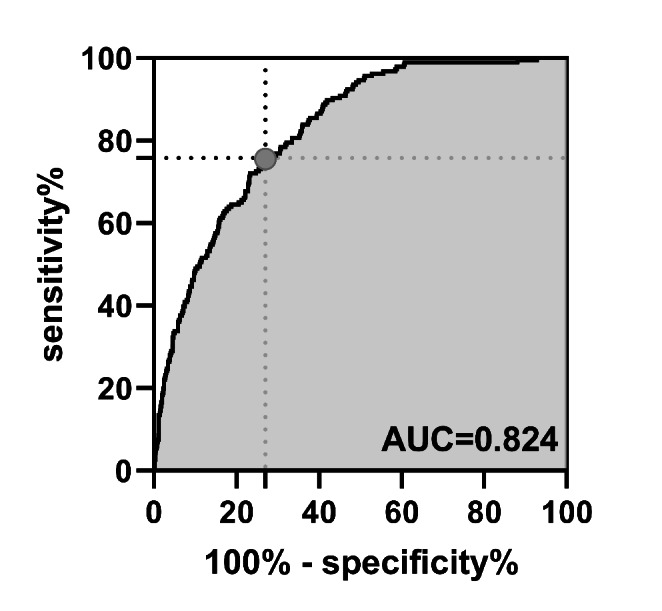
The ROC curve of the new model. The AUC of this new model was 0.824. The best cut-off value was the red dot with a sensitivity of 75.8% and a specificity of 73.0%. ROC: Receiver operating characteristic; AUC: Area under the curve

The regression coefficient (β) of the above logistic curve was used to construct a model to predict the risk of LAA thrombosis and SEC in NVAF patients: = 0.619*AF type + 0.654*previous stroke + 0.492*Fib + 0.9*LA − 0.64*LVEF + 0.214*LAA max caliber. The nomogram of this model can be seen in Fig. 3. The performance of the nomogram is test by the ROC curve, and the AUC = 0.824 (95% CI 0.797–0.851). We calculated the minimum Euclidean’s index and the optimal cut-off was determined with a sensitivity of 75.8% and a specificity of 73.0%. (Fig. 4).

## Discussion

The embolus of cardiogenic stroke mainly originates from the left atria and left atrial appendage. Thus, imaging markers of left atria system thrombus in patients with atrial fibrillation can be used as the evaluation criteria of hyperacute stroke [11]. Although several analyses have shown that cardiac computed tomography can avoid discomfort caused by TEE [12], TEE remains the mainstream method for LA thrombus examination [13]. Our research retrospectively analyzed 2591 TEE data of NVAF patients. There were 110 cases of left ventricular thrombus located in LAA, except for 1 case (0.9%) that was complicated with LA thrombus. The detection rate of LAA thrombosis was 4.2%, which is consistent with other literature [6]. Yoo et al. suggested that NVAF patients with SEC may develop a more severe stroke [14]. In this study, there were 103 s cases (4%) and 213 cases of thrombosis or SEC, accounting for 8.2% of patients in this cohort with a high risk of cardiogenic stroke. After combining two groups for regression analysis, it was found that the AF type, previous stroke, Fib, LA, LVEF, LAA max caliber were independent risk factors of LAA thrombosis and SEC in patients with NVAF. We constructed a combined prediction model by the results of regression and drew a nomogram, which can easily evaluate the future risk of LAA thrombosis and SEC in NVAF patients.

At present, the exact mechanism of LAA thrombosis in patients with NVAF is not quite sure. Based on our data and other literature, the mechanism may be related to various factors.

### Inflammatory reaction

Virchow suggested that abnormal blood stasis and abnormal hemostasis, platelets, and fibrinolysis may lead to endothelial or endocardial damage or dysfunction and, in turn, thrombus formation [8]. Experiments showed that rapid atrial pacing and atrial fibrillation state could cause inflammation, and the degree of inflammation in LA was greater than that of peripheral blood [15]. These inflammatory factors further lead to endocardial damage of LA and promote thrombosis. Our results showed that the NEUT in the thrombus group was significantly higher than in the control group (*p* < 0.001). Zotz et al. suggested that PLT activation and aggregation were also involved in the formation of thrombus [16]. Contrary, there was no significant difference in PLT between the two groups, which may be related to the activation location (data of our study came from peripheral blood, not LA blood).

### Chronic renal insufficiency

Elderly patients account for the majority of NVAF cases. They are often complicated with hypertension, CHD, diabetes and so on. These patients may have varying degrees of renal function damage. Kapłon-Cieślicka et al. suggested that chronic renal insufficiency should be regarded as an independent predictor of LAA thrombosis [17]. This study showed that the GFR in the thrombus group was lower than the control group (*P* < 0.001), while the Scr was significantly higher (*P* < 0.001), indicating that the control group had better renal function than the thrombus group.

### Abnormal coagulation and fibrinolytic

Several studies have shown that the dysfunction of coagulation and fibrinolysis system are important causes of thrombosis. Fibrinogen (Fib) can regulate erythrocyte aggregation in many ways; it can promote PLT aggregation through glycoprotein receptor complex [18], and induce thrombosis. In our study, Fib content in the thrombus group was higher than the control group (*P* < 0.001), and the differences of PT and APTT between the thrombus group and the control group were statistically different. Multivariate logistic regression analysis showed that Fib was an independent risk factor, indicating that LAA thrombosis in NVAF patients may be related to abnormal coagulation. Yet, there was no significant difference in TT between the two groups, indicating that the abnormal fibrinolytic system may not have a significant role in the process of LAA thrombosis.

### Structure and function of the heart

In AF patients, the atrial muscle loses its contractile function because of the disordered electrical activity of the atrial muscle [19, 20], which leads to slow and stasis blood flow in the atria. As a special part of LA, the anatomical structure of LAA makes it easier for blood to stagnate; thus, LAA is considered the main location of thrombosis [21]. Furthermore, larger LAA may lead to a lower blood emptying rate, resulting in longer blood stasis and promoting thrombosis [22]. In addition, the enlargement of LA, RA, and the decrease of LVEF are also important causes of thrombosis [23].

The result of this study showed that the diameter of LA and RA, the maximum caliber and depth of LAA in the thrombus group was significantly increased (*p* < 0.001), while the LVEF was significantly decreased compared with the control group (*p* < 0.001). Multivariate logistic regression analysis showed that the diameter of LA, the maximum caliber of LAA, and LEVF were independent risk factors.

### AF type

The correlation between the AF type and ischemic stroke is another important issue of clinical concern. It has been reported that left ventricular mass increase can predict LAA thrombosis in patients with persistent atrial fibrillation [24]. Atrial structural remodeling can accelerate the progression of AF and transform paroxysmal atrial fibrillation into persistent atrial fibrillation [20, 25]. In comparison to paroxysmal atrial fibrillation, persistent atrial fibrillation prolongs ineffective atrial ejection, which aggravates blood stasis and is more likely to lead to thrombosis [17]. The results of our study showed that the proportion of patients with persistent atrial fibrillation or permanent atrial fibrillation in the thrombus group was significantly higher than that in the control group (*p* < 0.001). Multivariate logistic regression analysis indicated that persistent or permanent atrial fibrillation was an independent risk factor. This indicates that patients who undergo persistent or permanent atrial fibrillation are more likely to have thromboembolic complications.

### LAA spontaneous echo contrast

Recent evidence has supported the view that LAA SEC is a prethrombotic state or hypercoagulable state [8]. Meus et al. suggested that SEC is a manifestation of blood stasis in LAA before thrombosis and the changes in blood inflammatory factors and coagulation factors before promoting thrombosis [26]. Boyd and colleagues further suggested that SEC is an independent and direct predictor of thrombosis in LAA [24]; SEC may be the intermediate state from normal blood flow to thrombosis in patients with NVAF. On the other hand, Tsai et al. indicated that SEC as LAA thrombus directly, and once SEC occurs, the blood emptying rate of LAA will decrease, which will accelerate the thrombosis [27].

In our study, patients with SEC were grouped separately and compared with the thrombus group and control group. The results showed that except for NEUT, LN, and HDL-c, all other variables in the SEC group were highly consistent with those in the thrombus group. Yet, there were no significant differences between the SEC group and control group or thrombus group while there was a significant difference between the control group and thrombus group, which indicates that SEC is a prethrombotic state of LAA, which may turn out to be thrombosis under the effect of inflammatory factors. Therefore, in clinical, SEC should be regarded as a very high-risk factor for thrombosis or the same as thrombosis, and anticoagulation therapy or interventional intervention (left atrial appendage occlusion) should be actively carried out, thus enabling patients to get a better prognosis.

## Limitation

The main limitation of our study is that compared with the control group, the number of thrombus group is too small, so we have to combine thrombus group and SEC group in regression analysis to increase the accuracy of the regression model. The result is a series of independent risk factors of “high cardiogenic stroke risk”, including the risk of LAA thrombosis and severe SEC, instead of evaluating the occurrence of LAA thrombosis straightforwardly. Second, although the types of atrial fibrillation were classified, we do not record the total time of the atrial fibrillation state in NVAF patients in this study, so the specific burden of atrial fibrillation on patients could not be evaluated. Third, previous studies have also shown that D-dimer is associated with a variety of thromboembolic complications. Moreover, some studies have shown that D-dimer can be used as one of the indicators for predicting LAA thrombosis [28]. It was the limitation of the present study, we did not analysis it because of too much D-dimer data missing. And we do not include lobe number in analysis because of the incomplete data of LAA morphology, which is one of the limitations of our study.

## Conclusion

In patients with NVAF who underwent TEE, the detection rate of LAA thrombosis was 4.2%. AF type, previous stroke, Fibrinogen, diameters of the left atria, left ventricular ejection fraction, and LAA maximum caliber resulted as strong, independent predictors for (LAA thrombosis and severe SEC and we can evaluate the future criticality of cardiogenic stroke risk (LAA thrombosis and severe SEC) by using our effective nomogram.
